# Long working hours and health

**DOI:** 10.1016/j.lanwpc.2021.100199

**Published:** 2021-06-25

On May 17, 2021, WHO and the International Labour Organization (ILO) released the WHO/ILO Joint Estimates of the Work-related Burden of Disease and Injury (WHO/ILO Joint Estimates), published in the journal Environment International. According to the report, 488 million people worldwide have long working hours, and more than 745 000 people died in 2016 from heart disease and stroke related to working more than 55 hours per week. These figures make long working hours one of the biggest occupational health hazards.

The health consequences of long working hours vary depending on factors such as the length of the working hours, job characteristics, socioeconomic status, and the individual's health condition. Overworking increases the risk of cardiovascular diseases and stroke, as well as affecting mental health. Physical and mental strain of the overworked people can start from acute physiological responses such as fatigue, stress, impaired sleep, and unhealthy lifestyle changes in response to the stress. In addition, overworking reduces work performance and results in productivity loss due to illness and occupational injuries.

Since the ILO put the 8-hour workday into international law in 1919, the average working time has decreased in most countries globally, but the number of people who overwork has been increasing since 2000 according to the WHO/ILO Joint Estimates. The uptrend is more frequently observed in people living in the Western Pacific and southeast Asia regions. Many countries in these regions have the longest working hours compared with the rest of the world. For example, in South Korea, 25·2& of employees work at least 50 hours per week, compared with the average of 11& in member countries of the Organisation for Economic Cooperation and Development. Overworking is so common in Japan that there is a legal term, *karoshi*, which means death by overwork, to describe the cause of death. The 2018 report on World Employment and Social Outlook released by the ILO showed that people in low-income and middle-income countries were inclined to work longer than those in high-income countries; thus, it is unsurprising that the overworked phenomenon is even more common in the region's lower-income countries.

Long working hours have been praised for contributing to fast economic growth in the Asia-Pacific countries. By contrast, the side-effects of overworking have been discussed but have not received sufficient attention. Unlike other working conditions, which might involve exposures that are difficult to avoid due to the nature of the job, working time can be modified through legal amendments; however, the reality is much more complex. Despite years of labour reforms to reduce the length of the working day, overworking remains a serious social issue in many countries in the Western Pacific region. Other than social and cultural factors, capitalism's demand for profit as well as financial concerns and fears over job insecurity from the perspective of the workforce drive people to work more. The rise of the so-called gig economy and telework was supposed to enable reconciliation between work and daily life, but rather than the convenience and flexibility it has provided, the new work model has been criticised for blurring work and life boundaries and raising demands and expectations from employers. The COVID-19 pandemic is likely to be worsening the situation. A study of 3·1 million employees from North America, Europe, and the Middle East in 2020 suggested that the average working hours had increased by 48·5 minutes. For health workers and other essential workers, the growing demands for their services to maintain daily life has made them work for a prolonged period during the pandemic.

Long working hours have been on the rise for decades, resulting in an increasing number of people developing health consequences related to work pressure. The WHO/ILO Joint Estimate is a wake-up call regarding this unhealthy yet under-recognised practice and highlights the urgent need to address this health crisis. The solution is likely to involve change and collaboration between employers and employees, and more importantly, fundamental adjustments at governmental and legislative levels to adopt and enforce labour standards on working hours. After all, prevention is a more cost-effective option to resolve this social and health issue.

*The Lancet Regional Health – Western Pacific*

